# Analgesic effects of erector spinae plane block can differ according to needle size: A case report

**DOI:** 10.1097/MD.0000000000027142

**Published:** 2021-09-03

**Authors:** Hobum Cho, Jiwon Chung, Younsil Jang, Sanghoon Song, Jaehwa Yoo, Sangho Kim, Sunyoung Park, Mungyu Kim

**Affiliations:** Department of Anesthesiology and Pain Medicine, Soonchunhyang University Hospital Seoul, Seoul, Republic of Korea.

**Keywords:** erector spinae plane block, paralysis, phrenic nerve, postoperative pain

## Abstract

**Rationale:**

Unlike brachial plexus block, erector spinae plane block (ESPB) does not target specific nerves, so the analgesic effect may differ depending on the extent of diffusion of local anesthetic. Therefore, needle size, which can affect the diffusion of local anesthetic, may be an important factor in the analgesic effect.

**Patient concerns:**

Four patients with end-stage renal disease on hemodialysis received vascular surgery due to arteriovenous fistula occlusion. Vascular bypass surgery was performed on the axillary vein.

**Diagnoses:**

Four patients with end-stage renal disease on hemodialysis were diagnosed with arteriovenous fistula occlusion. One in 4 patients was diagnosed with diaphragm paralysis after ESPB, and the other 3 did not develop diaphragm paralysis.

**Interventions:**

ESPB was conducted by ultrasound using a 25- or 22-gauge needle at the C7 level. The extent of nerve blockade was determined based on cold sensation, and diaphragm excursion and thickness were measured via ultrasound.

**Outcomes:**

The analgesic effect was excellent in 2 patients treated using a 22-gauge needle, but was poor in 2 other patients treated with a 25-gauge needle.

**Lessons:**

ESPB at the C7 level can cause diaphragm paralysis, and needle size may affect the extent of diffusion of local anesthetic.

## 1 Introduction

Erector spinae plane block (ESPB) at the T2–3 level can be effective for acute and chronic shoulder pain because the local anesthetic can reach the cervical nerve root at the C3–4 level.^[1,2]^ We previously reported that ESPB at the T2 level may be effective for postoperative management of patients undergoing arm surgery.^[3]^ However, the pattern of cephalad diffusion of local anesthetic at the T2 level was poorly reproducible. Therefore, we performed ESPB at the C7 level to enhance cephalad diffusion of local anesthetic, and observed the effects and complications. We report this case because we accidentally found that the extent of cephalad diffusion may vary by needle size.

## 2 Methods

We recommended ESPB to 4 patients undergoing vascular bypass surgery of the axillary vein due to arteriovenous fistula occlusion and performed it after obtaining informed consent.

**Figure 1:**
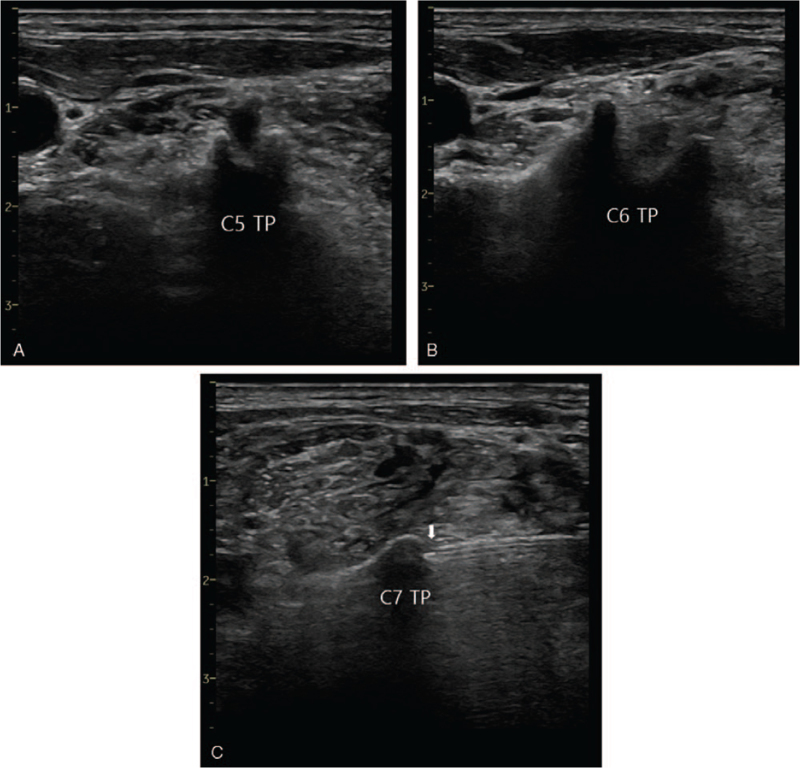
(A), (B) The C5 and C6 transverse processes, which have an anterior and posterior tubercle, respectively. (C) The C7 transverse process, which has no anterior tubercle; the white arrow indicates the needle tip. TP = transverse process.

ESPB was conducted in the lateral position using ultrasound (Logiq P6; GE Healthcare, Chicago, IL). The ipsilateral C7 transverse process, which has no anterior tubercle, and the C5 and C6 transverse processes, which have an anterior and posterior tubercle, respectively, were checked. A 22-gauge Quincke needle or 6 cm 25-gauge needle was inserted from the posterior to anterior direction, toward the posterior tubercle of the C7 transverse process. After the needle contacted the posterior tubercle of the C7 transverse process, 25 mL 0.45% ropivacaine was injected (Fig. 1).

## 3 Case presentation

### 3.1 Case 1

A 69-year-old woman was taking medication for hypertension and diabetes mellitus. She started hemodialysis 9 years previously and underwent 2 thrombectomies (1 and 2 years ago). She was scheduled for vascular bypass surgery of the axillary vein in the left arm under general anesthesia. She requested intravenous patient-controlled analgesia because of severe pain during the last surgery, and we recommended ESPB for postoperative pain control prior to general anesthesia. We explained to her that ESPB can cause temporary muscle weakness in the ipsilateral upper limb and diaphragmatic paralysis after surgery, and obtained her consent.

**Figure 2:**
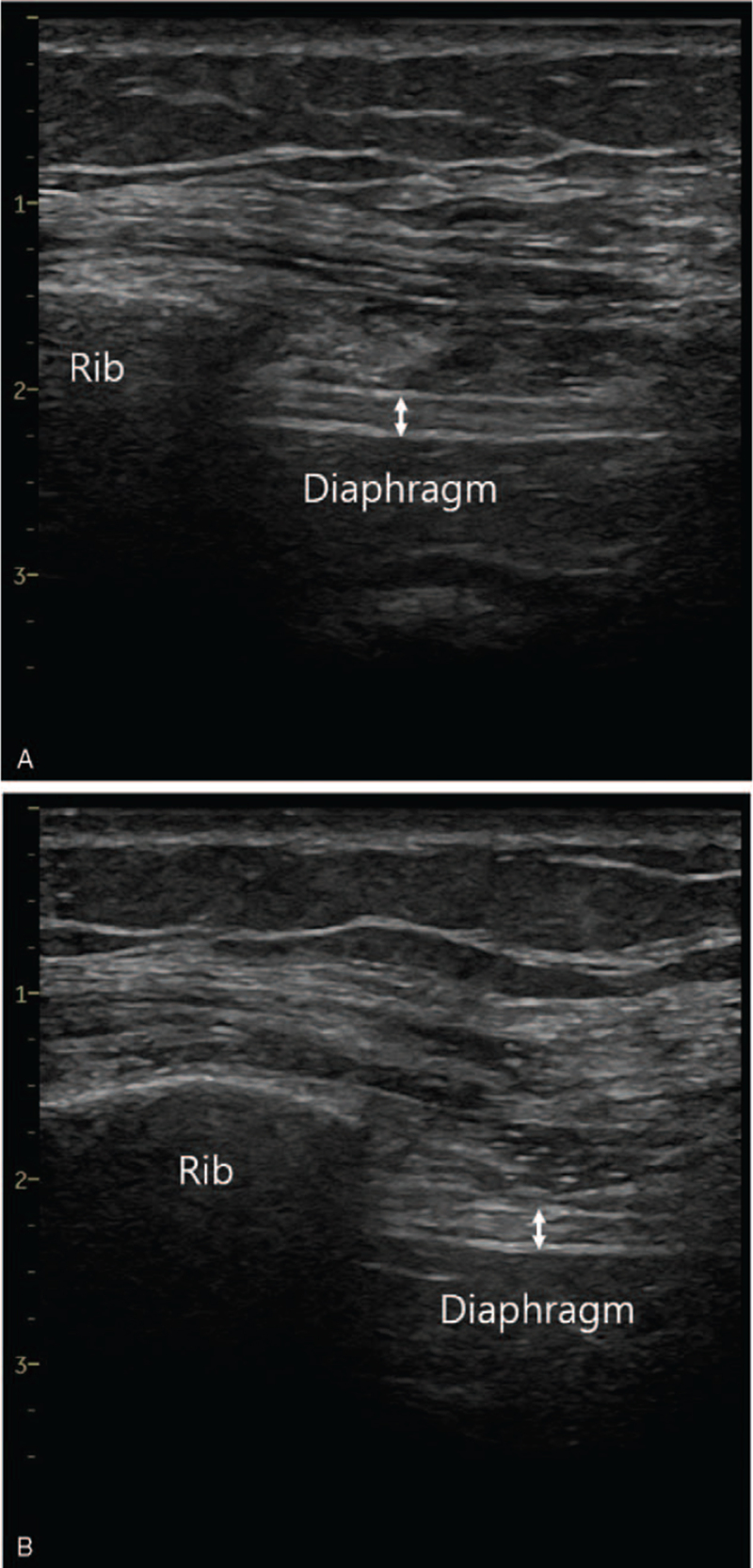
There was little difference in diaphragm thickness between maximal inspiration and end expiration. (A) At end expiration, the diaphragm thickness was 2.0 mm. (B) At maximal inspiration, the diaphragm thickness was 2.1 mm.

ESPB was performed using a 22-gauge Quincke needle in the manner described above. After ESPB, general anesthesia was performed using propofol and remifentanil. The anesthesia time was 75 minutes. In the recovery room, the extent of nerve blockade was determined based on cold sensation from C3 to T2. It was almost impossible for the patient to bend her wrist and elbow. The numeric rating scale (NRS) pain score (range: 0–10) was 0 and no painkillers were administered. Diaphragm paralysis was assessed using ultrasound. The probe was placed in the ipsilateral subcostal area, between the anterior axillary and midclavicular line. Diaphragm movement during deep breathing was not clearly visible in M-mode. Diaphragm thickness was measured at the anterior axillary line, between the 7th and 8th intercostal space. The diaphragm thickness was similar between maximal inspiration and end expiration, at 2.1 and 2.0 mm, respectively (Fig. 2). Although the patient had no respiratory symptoms, she was diagnosed with ipsilateral diaphragm paralysis. No respiratory complications were observed in the chest X-ray (PA view) conducted on POD 1 and she was discharged on POD 2.

### 3.2 Case 2

A 57-year-old man who has been on hemodialysis for 13 years underwent 5 vascular operations in both arms. He was scheduled for vascular bypass surgery of the right arm. ESPB was performed at the C7 level using a 22-gauge Quincke needle, and then general anesthesia was induced by total intravenous anesthesia. The anesthesia time was 65 minutes. In the recovery room, the extent of sensory nerve blockade was measured between the C4 and T1 dermatomes. Elbow flexion and extension were possible, but the muscle strength was reduced to grade 3. Diaphragm excursion during sniffing was measured at 2.95 cm, indicating that diaphragm paralysis was unlikely. The NRS pain score was 1 and no analgesics were given.

### 3.3 Case 3

A 63-year-old woman was scheduled for vascular bypass surgery of the left arm. ESPB was administered in the same manner as in the previous cases, but a 6 cm 25-gauge needle was used to reduce needle pain. After ESPB, general anesthesia was performed (total intravenous anesthesia). The anesthesia time was 70 minutes and nerve blockade was effective at the C8–T1 level. Movement of the arm was not restricted on the surgical side and the strength was the same as the opposite side. Upon sniffing, diaphragm excursion was measured at 2.36 cm. The NRS pain score was 5. After administering 20 μg fentanyl, the pain was slightly reduced to an NRS score of 4. The patient complained of nausea and refused additional analgesics.

### 3.4 Case 4

A 76-year-old man was scheduled for vascular bypass surgery of the left arm. ESPB was administered in the same way as in patient 3, that is, using a 6 cm 25-gauge needle. The general anesthesia time was 75 minutes. In the recovery room, no reduction in cold sensation was observed in any areas on the surgical side and the muscle strength was similar to that of other side. During sniffing, the diaphragm excursion was 2.89 cm. The patient complained of severe pain (NRS pain score of 7) and was given 2 20 μg fentanyl doses with a 15 minutes interval. His pain decreased to an NRS pain score of 3. Ramosetron 0.3 mg was administered before the patient left the recovery room due to nausea.

## 4 Discussion

Vascular surgery for hemodialysis in patients with end-stage renal disease is mostly performed in the forearm under local anesthesia. However, in cases of re-operation due to arteriovenous fistula occlusion, the brachial or axillary vessels can be used for the vascular bypass surgery. In particular, if performed using the axillary vein, general anesthesia may be required for vascular surgery because the axillary region is not anesthetized by brachial plexus block. Therefore, we performed ESPB at the C7 level to reduce postoperative pain during vascular surgery requiring general anesthesia.

Since 2016, many studies have reported that ESPB has a postoperative analgesic effect after abdominal and thoracic surgery, but few studies have evaluated its use in upper limb and shoulder surgery.^[4–6]^ In addition, studies on diaphragm paralysis, which can occur after ESPB at the cervical level, remain scarce. Diwan and Nair^[2]^ reported that ESPB performed at the T2–3 level had an excellent pain-relieving effect in patients who underwent open fixation of the proximal humerus, along with diminished cold sensation between the C3 and T3 dermatomes. They suggested that ESPB could serve as an alternative method for diaphragm-sparing block during shoulder surgery. Ma et al^[7]^ reported that high thoracic ESPB provided an adequate analgesic effect after proximal humerus surgery and total shoulder arthroplasty without phrenic nerve blockade. However, in a cadaver study, when 20 mL dye mixture was injected at the C6 or C7 level, the phrenic nerve was stained by 3 of 10 injections.^[8]^ In our case, phrenic nerve and brachial plexus block were observed in 1 (case 1) of 2 patients who had an excellent analgesic effect. For this reason, we believe that ESPB at the C7 level may be more likely to cause diaphragmatic paralysis.

Diaphragmatic paralysis can be evaluated by measuring diaphragm excursion or thickness. In men, the average diaphragm excursion during sniffing has been reported to be 2.9 to 3.1 cm, with a lower limit of 1.8 to 1.9 cm. In women, the respective values were 2.6 to 2.7 and 1.6 to 1.7 cm.^[9]^ If the diaphragm thickening ratio between end expiration and maximal inspiration is less than 1.2, diaphragm dysfunction can be diagnosed.^[10]^ In case 1 in our study, diaphragm excursion was less than 1 cm during deep breathing and there was little difference in diaphragm thickness between maximal inspiration and end expiration. We diagnosed her with diaphragmatic paralysis. In case 2, diaphragm excursion during sniffing was higher than the lower limit. However, partial diaphragm paresis could not be completely ruled out because we did not conduct a diaphragm examination before ESPB.

Unlike brachial plexus block, ESPB does not target specific nerves, so the analgesic effect may differ depending on the extent of local anesthetic diffusion. We used a 6 cm 25-gauge needle to reduce needle pain in cases 3 and 4. The smaller the needle size, the higher the pressure when injecting the local anesthetic and the higher pressure can push the needle back. Therefore, it may be difficult to inject the local anesthetic into the correct location at once, and the high pressure may limit the diffusion of the local anesthetic. In our results, the analgesic effect was excellent in 2 patients (cases 1 and 2) treated using the 22-gauge needle, but there was little to no analgesic effect in the 2 patients (cases 3 and 4) treated with the 25-gauge needle. Therefore, needle size appears to have a significant impact on the diffusion of local anesthetics.

In conclusion, although ESPB at the C7 transverse process is effective for relieving pain after upper limb surgery, it can cause diaphragmatic paralysis. In addition, a small needle may limit cephalad diffusion of local anesthetic. Therefore, ESPB should be carried out with a needle size larger than 22-gauge whenever possible. Further studies on the relationship between ESPB and diaphragm paralysis, and on the most appropriate needle size for diffusion of local anesthetic, are needed.

## Author contributions

**Conceptualization:** Jiwon Chung, Mun Gyu Kim.

**Data curation:** Younsil Jang, Jaehwa Yoo.

**Investigation:** Sanghoon Song.

**Supervision:** Sangho Kim.

**Writing – original draft:** Hobum Cho, Sunyoung Park.

**Writing – review & editing:** Mun Gyu Kim.
